# Persistent Organochlorine Pollutants with Endocrine Activity and Blood Steroid Hormone Levels in Middle-Aged Men

**DOI:** 10.1371/journal.pone.0066460

**Published:** 2013-06-13

**Authors:** Elise Emeville, Frank Giton, Arnaud Giusti, Alejandro Oliva, Jean Fiet, Jean-Pierre Thomé, Pascal Blanchet, Luc Multigner

**Affiliations:** 1 Inserm U1085, Irset, Pointe à Pitre, Guadeloupe, French West Indies; 2 AP-HP CIB GHU Sud, Hospital Henri Mondor, Créteil, France; 3 Inserm U955, Eq07, Centre de Recherches Chirurgicales, Créteil, France; 4 Center for Analytical Research and Technology, Liege University, Liege, Belgium; 5 Programa de Medio Ambiente y Salud, Universidad Nacional de Rosario, Rosario, Argentina; 6 Service d'Urologie, Centre Hospitalier Universitaire Guadeloupe, Pointe à Pitre, Guadeloupe, French West Indies; University of North Carolina at Chapel Hill, United States of America

## Abstract

**Background:**

Studies relating long-term exposure to persistent organochlorine pollutants (POPs) with endocrine activities (endocrine disrupting chemicals) on circulating levels of steroid hormones have been limited to a small number of hormones and reported conflicting results.

**Objective:**

We examined the relationship between serum concentrations of dehydroepiandrosterone, dehydroepiandrosterone sulphate, androstenedione, androstenediol, testosterone, free and bioavailable testosterone, dihydrotestosterone, estrone, estrone sulphate, estradiol, sex-hormone binding globulin, follicle-stimulating hormone, and luteinizing hormone as a function of level of exposure to three POPs known to interfere with hormone-regulated processes in different way: dichlorodiphenyl dichloroethene (DDE), polychlorinated biphenyl (PCB) congener 153, and chlordecone.

**Methods:**

We collected fasting, morning serum samples from 277 healthy, non obese, middle-aged men from the French West Indies. Steroid hormones were determined by gas chromatography-mass spectrometry, except for dehydroepiandrosterone sulphate, which was determined by immunological assay, as were the concentrations of sex-hormone binding globulin, follicle-stimulating hormone and luteinizing hormone. Associations were assessed by multiple linear regression analysis, controlling for confounding factors, in a backward elimination procedure, in multiple bootstrap samples.

**Results:**

DDE exposure was negatively associated to dihydrotestosterone level and positively associated to luteinizing hormone level. PCB 153 was positively associated to androstenedione and estrone levels. No association was found for chlordecone.

**Conclusions:**

These results suggested that the endocrine response pattern, estimated by determining blood levels of steroid hormones, varies depending on the POPs studied, possibly reflecting differences in the modes of action generally attributed to these compounds. It remains to be investigated whether this response pattern is predictive of the subsequent occurrence of disease.

## Introduction

An endocrine-disrupting chemical (EDC) is an exogenous chemical that can interfere with various aspects of hormone function, synthesis, secretion, regulation, action and elimination [1], [2]. EDCs may thus have deleterious effects on many endocrine system and outcomes in both humans and wildlife [3]. There is growing evidence that adverse reproductive outcomes, including reproductive organ tumors, may result from exposure to EDCs present at low concentrations in the environment, although epidemiological evidence of a causal relationship remains limited [4].

Various EDCs exert their effects through steroid-mediated pathways, by interfering with the binding of physiological ligands to steroid receptors and binding proteins and enzymes involved in the steroid biosynthesis pathway [5]. The synthesis and secretion of steroid hormones are controlled by positive and negative feedback mechanisms, but it has been suggested that exposure to EDCs may also result in slight, but real modifications of circulating steroid hormone levels.

Several studies have investigated relationships between persistent organochlorine pollutants (POPs) with endocrine properties and a limited number of steroid hormones, mostly testosterone (T) and estradiol (E2), in blood samples from populations of adult men. These studies have focused mostly on ubiquitous environmental pollutants, such as dichlorodiphenyl dichloroethene (DDE, the major and most stable metabolite of dichlorodiphenyl trichloroethane, DDT) and polychlorinated biphenyls (PCBs) [6]–[22]. No significant effect was found in most of these studies, but the overall picture is not uniform. There are several possible reasons for these discrepancies and for the lack of comparability between studies: differences in the age range investigated or in the exposure levels experienced by the populations, lack of controls for some potentially confounding factors and the use of different immunological hormone assay methods with different performances.

We investigated the possible effects of long-term exposure to various POPs on blood levels of steroid hormones, binding proteins and gonadotrophins in healthy, non obese, middle-aged French West Indian men. We focused on DDE, PCBs and chlordecone. These chemicals are known to bind to androgen (AR) and/or estrogen (ER) steroid receptors and to interfere with hormone-regulated processes in different ways [23]–[25]. The effects of these compounds on blood steroid levels may be mediated by effects on any of the components of the steroid pathway. We therefore investigated a wide panel of blood androgens and estrogens, determining the levels of these compounds mostly by gas chromatography-mass spectrometry (GC–MS), the gold standard method for steroid hormone assay [26], [27]. Given the interconnection between steroid production by the testis and hypophyseal hormones, we also determined circulating follicle-stimulating hormone (FSH) and luteinizing hormone levels (LH).

## Materials and Methods

### Ethics Statement

The study was approved by the Guadeloupe Ethics Committee for studies involving human subjects. Each participant provided written informed consent.

### Study Population

This study took place in Guadeloupe (French West Indies), a Caribbean archipelago, most of the inhabitants of which are of African descent. Subjects were recruited from men participating in a free yearly systematic health-screening program funded by the French national health insurance system. Each year, a random sample of the population, selected so as to be representative of the age and sex distribution of the general population, is invited to participate in the program at a single site. As part of the Karuprostate prostate cancer study, consecutively enlisted men aged 45 to 69 years of age were invited to participate [28]. The acceptance rate was around 90%. Information was obtained from participants about their demographic characteristics, anthropometric measurements, lifestyle, medical records and medication use. The inclusion criteria for this study were: a) both parents born on any Caribbean island with a population of predominantly African descent, b) no history of a chronic medical disorder and standard biochemical and hematological blood parameters in the normal range, c) no hormone treatments or drugs known to influence the hypothalamic-pituitary-gonadal-adrenal axis (including inhibitors of 5 α reductase), d) body mass index (BMI)<30. A blood sample was drawn from each participant between 8.00 and 10.00 a.m., after overnight fasting. Serum samples were separated and frozen at −30° until shipment. Samples were identified only by a unique sample code and were transferred by airmail, on dry ice, to Creteil (France) for hormone analysis and to Liege (Belgium) for organochlorine and lipid analysis. All laboratory personnel were blind to the identities of subjects.

Given the high cost of the analytical methods used, we limited the initial sample size to 300 individuals, which, for a statistical power of 0.8 and a *p* level of 0.05 gave an anticipated minimum effect size (f^2^) of 0.05 for multiple regression studies with eight predictors, not including the regression constant. We randomly selected 60 individuals for each five-year age group, from the age of 45 to 69 years. We excluded the men who did not fulfill the inclusion criteria or who had provided too little blood to carry out all the hormonal and chemical assays, resulting in a final sample size of 277 subjects.

### Measurement of hormones

Dehydroepiandrosterone (DHEA), androstenedione (AD), androstenediol (ADIOL), total testosterone (T), dihydrotestosterone (DHT), estrone (E1), E2, and estrone sulfate (E1S) were assayed simultaneously by GC-MS, as previously described [29]. Briefly, deuterated steroid internal standards (CDN Isotopes, Inc., Point-Claire, Quebec, Canada) were added to all serum samples, which were then extracted with 1-chlorobutane. The organic extracts were purified on conditioned high-purity silica LC-Si SPE columns (Varian, Les Ulis, France). All steroids were derivatized with pentafluorobenzoyl chloride, except for AD, which was derivatized with pentafluorobenzylhydroxylamine. The final extracts were reconstituted in isooctane, then transferred to conical vials for injection into the GC system (6890N, Agilent Technologies, Massy, France), equipped with a 50% phenylmethylpolysiloxane VF-17MS capillary column (20 m×0.15 mm, internal diameter, 0.15 mm film thickness; Varian). An HP5973 (Agilent Technologies) quadrupole mass spectrometer equipped with a chemical ionization source and operating in single-ion monitoring mode was used for detection. E1S was determined as E1 after acid solvolysis [30]. Free T (fT) concentrations were calculated from T and SHBG concentrations, as previously described [31]. Bioavailable T (BT) concentration was determined as described elsewhere [29], [31]. DHEAS was determined by a radioimmunological method (IM 0729, Beckman Coulter, Marseilles, France). Plasma SHBG, FSH and LH levels were determined by radioimmunometric methods (using the following kits: Schering SHBG RIACT, Gif-sur-Yvette, France; FSH kit and LH kit, Coulter Immunotech, Marseilles, France). The molecular masses of the derivatized steroids (deuterated and corresponding non deuterated steroids), as assayed by GC–MS, and the means and intra- and inter-assay coefficients of variation of four quality control sera (one with very low concentrations of the assayed steroids for determination of the lower limit of quantification by GC-MS, and three with higher concentration levels) are reported in Supporting Information File S1 (Table S1). The results of BT, DHEAS, SHBG, FSH and LH quality controls are also reported in Table S1.

### Measurement of organochlorines

A high-resolution gas chromatograph (Thermo Quest Trace 2000, Milan, Italy) equipped with a Ni63 electron capture detection system was used to assess the serum concentrations of seven indicator PCB congeners (28, 52, 101, 118, 138, 153, and 180); p,p′-DDT, p,p′-dichlorodiphenyl dichloroethane (DDD) and p,p′-DDE; the α, β, and γ isomers of hexachlorocyclohexane (HCH) and chlordecone. The limits of detection (LD) were 0.05 µg/l for all organochlorine compounds except chlordecone (0.06 µg/l). Detailed information about sampling, analysis and quality assurance and control have been provided elsewhere [28], [32]. Plasma total cholesterol and total triglyceride concentrations were determined enzymatically (DiaSys Diagnostic Systems GmbH; Holzheim, Germany) and total lipid concentration was calculated as previously described [33].

### Data and statistical analysis

Continuous measurements were described in terms of the median, range, mean and percentiles. We restricted our analysis to pollutants with a detection frequency greater than 80%: DDE (97%), PCB congeners 138 (96%), 153 (98%) and 180 (97%) and chlordecone (87%). Values below the LD were estimated by a maximum likelihood estimation method [34]. Correlations between the concentrations of frequently detected pollutants were explored by Spearman's rank correlation analysis (Supporting Information File S1, Table S2). The concentrations of the various PCBs were highly correlated, so we restricted further analysis to PCB 153.

The effects of exposure variables on the outcome variables were evaluated by linear regression analyses. Scatter plots were used for bivariate comparisons between exposure and outcome variables, to ensure that linear associations were reasonable. Model assumptions were checked by residual analysis. A normal distribution of the residuals was achieved by natural log transformations of outcomes and hormone ratios (DHEA, DHEAS, T, AD, E2, E1, E1S, LH, FSH, and T/E2) or by square root transformations (ADIOL, fT, BT, SHBG, and T/LH). The exposure variables were treated as continuous variables and were subjected to natural log transformation. The regression coefficient (β) represented the change of naturally-log or square root transformed outcomes variables per one unit of naturally-log transformed serum organochlorine concentration The following covariates were considered as potential confounding factors: age (years, as a continuous variable), body mass index (BMI) (kg/m^2^), waist-to-hip ratio (continuous), alcohol (current or past alcohol use vs never) and tobacco consumption (current or past smoking vs never), education (elementary/middle/high school), season of blood collection (spring, summer, autumn, winter) and total lipid concentration (mg/dl). For each exposure predictor, we also considered the other contaminants as potential confounders. A parsimonious and stable regression model was obtained, by the use of a data-dependent procedure based on backward variable elimination (PROC GLMSELECT) in multiple bootstrap samples [35], [36]. Variables were retained if they were selected in at least 30% of the 1000 bootstrap samples with α = 0.05. All analyses were carried out with SAS software version 9.3 (SAS Institute, Inc., Cary, NC, USA). All tests were two-tailed, and *p* values≤0.05 were considered statistically significant.

## Results

The participants were Caribbean men of African descent aged between 45 and 69 years (median: 58 years). Overall, 61% had never smoked and 15% were teetotal. The characteristics of the study population with respect to potential confounders and outcome variables are given in Table 1. Detection and concentrations of POPs in the serum samples of the study population are presented in Table 2

**Table 1:** Characteristics of the study population in terms of potential confounders and outcome variables.

**Potential confounders**	
Age (years)^a^	58.3, 58.1 (45.1–69.9)
Education (%), high school, middle school, elementary school	9.3, 37.4, 53.3
BMI (kg/m^2^)^a^	24.7, 24.9 (17.9–29.8)
Waist-to-hip ratio^a^	0.90, 0.90 (0.72–1.29)
Current or past alcohol use (%)	84.8
Current or past smoking (%)	38.6
Total lipids (mg/dl)^b^	5.6 (3.7–8.6)
Season of blood sampling (%) summer, autumn, winter, spring	24.1, 29.0, 24.9, 22.0
**Outcome variables** b **^, ^** c	
DHEA (nmol/l)	9.8 (4.3–19.8)
DHEAS (µmol/l)	2.8 (1.0–6.9)
AD (nmol/l)	4.0 (2.4–7.7)
ADIOL (nmol/l)	5.3 (1.5–12.4)
E1 (pmol/l)	152.2 (89.1–282.2)
E1S (nmol/l)	1.6 (0.6–4.5)
E2 (pmol/l)	110.1 (60.6–189.3)
T (nmol/l)	18.1 (9.4–31.4)
fT (nmol/l)	0.33 (0.16–0.61)
BT (nmol/l)	6.5 (3.4–10.6)
DHT (nmol/l)	1.9 (0.8–4.1)
SHBG (nmol/l)	35.2 (13.1–73.8)
FSH (IU/l)	6.6 (2.2–24.3)
LH (IU/l)	4.9 (2.0–12.0)

a Values are mean, median (minimum – maximum).

b Values are mean (5^th^–95^th^ percentiles).

c Back-transformed values.

Abbreviations, DHEA: dehydroepiandrosterone; DHEAS: dehydroepiandrosterone sulfate; AD: androstenedione; ADIOL: androstenediol; E1: estrone; E1S: estrone sulfate; E2: estradiol; T: testosterone; fT: free testosterone; BT: bioavailable; DHT: dihydrotestosterone; SHBG: sex hormone binding protein; FSH: follicle-stimulating hormone; LH: luteinizing hormone.

**Table 2:** Detection and concentrations (µg/l) of persistent organochlorine pollutants in serum samples of the study population.

Organochlorine	Detection frequency	Geometric mean	Min	Percentiles					Max
	(%)	(µg/l)	(µg/l)	(µg/l)					(µg/l)
				10^th^	25^th^	50^th^	75^th^	90^th^	
p,p′- DDT	34.9	-	<LD	<LD	<LD	<LD	0.06	0.17	1.71
p,p′- DDD	27.5	-	<LD	<LD	<LD	<LD	0.06	0.10	0.79
p,p′- DDE	96.9	1.77	<LD	0.38	0.96	2.06	4.03	7.22	27.4
PCB 28	51.0	-	<LD	<LD	<LD	0.05	0.22	0.64	2.97
PCB 52	38.0	-	<LD	<LD	<LD	<LD	0.20	0.57	3.02
PCB 101	58.0	-	<LD	<LD	<LD	<LD	0.15	0.25	0.62
PCB 118	59.6	-	<LD	<LD	<LD	<LD	0.19	0.38	4.64
PCB 138	96.1	0.50	<LD	0.16	0.33	0.53	0.94	1.45	4.12
PCB 153	98.5	0.75	<LD	0.21	0.49	0.87	1.48	2.32	6.46
PCB 180	96.8	0.64	<LD	0.24	0.42	0.67	1.08	1.66	5.52
α - HCH	39.6	-	<LD	<LD	<LD	<LD	0.09	0.15	1.20
β - HCH	41.6	-	<LD	<LD	<LD	<LD	0.08	0.12	0.69
γ - HCH	38.0	-	<LD	<LD	<LD	<LD	0.08	0.14	1.12
Chlordecone	86.7	0.40	<LD	<LD	0.20	0.45	0.95	1.74	44.1

Abbreviations, LD: limits of detection; DDT: dichlorodiphenyl trichloroethane; DDD: dichlorodiphenyl dichloroethane; DDE: dichlorodiphenyl dichloroethene; PCB: polychlorinated biphenyl; HCH: hexachlorocyclohexane.

The results of linear regression analysis for continuous exposure variables are given in Table 3 for DDE, Table 4 for PCB 153, and Table 5 for chlordecone. For each exposure, we present a crude nonadjusted model and a backward adjusted model coupled to bootstrap selection covariates.

**Table 3:** Regression coefficients (β) for association between serum pp′ DDE levels and the outcome variables.

Outcomes	Crude		Adjusted	
	β	95% CI (β)	β	95% CI (β)
ln DHEA (nmol/l)	−0.027	−0.076 to 0.021	−0.021^a^^,^ b	−0.067 to 0.026
ln DHEAS (µmol/l)	−0.027	−0.081 to 0.027	−0.020^a^^,^ c	−0.073 to 0.033
ln AD (nmol/l)	0.014	−0.021 to 0.050	0.010^a^^,^ c ^–^ e ^,^ g	−0.026 to 0.045
sqrt ADIOL (nmol/l)	−0.017	−0.080 to 0.047	−0.010^a^^–^ c ^,^ e	−0.071 to 0.050
ln E2 (pmol/l)	0.022	−0.008 to 0.052	0.018^c^	−0.012 to 0.047
ln E1 (pmol/l)	0.016	−0.018 to 0.050	0.004^c^^,^ g	−0.030 to 0.039
ln E1S (nmol/l)	0.052	−0.009 to 0.112	0.037^d^^,^ h	−0.023 to 0.097
ln T (nmol/l)	−0.010	−0.043 to 0.023	−0.003^c^^,^ d	−0.036 to 0.030
sqrt fT (nmol/l)	0.003	−0.008 to 0.014	0.007^a^^,^ g ^,^ i	−0.003 to 0.017
sqrt BT (nmol/l)	−0.003	−0.044 to 0.037	0.013^a^^,^ c ^,^ g	−0.027 to 0.053
ln DHT (nmol/l)	−0.062*	−0.109 to −0.015	−0.063* a ^,^ c ^–^ f	−0.109 to −0.016
sqrt SHBG (nmol/l)	−0.099	−0.240 to 0.041	−0.102^a^^,^ d ^,^ f ^,^ i ^,^ j	−0.230 to 0.026
ln LH (IU/l)	0.053*	0.001 to 0.106	0.057* a ^–^ d ^,^ h	0.005 to 0.109
ln FSH (IU/l)	0.023	−0.042 to 0.088	0.001^a^^,^ c ^,^ j	−0.063 to 0.065
sqrt T/LH	−0.066*	−0.118 to–0.013	−0.061* a ^–^ c ^,^ h	−0.112 to −0.010
ln T/E2	−0.032	−0.065 to 0.0009	−0.022^c^^–^ e ^,^ g	−0.054 to 0.010

a : age;

b : alcohol;

c : season of blood sampling;

d : BMI;

e : education;

f : chlordecone;

g : PCB-153;

h : smoking;

i : waist-to-hip-ratio;

j : blood total lipids.

* Statistically significant association because 95% CI (β) does not include zero.

**Table 4:** Regression coefficients (β) for association between serum PCB-153 levels and the outcome variables.

Outcomes	Crude		Adjusted	
	β	95% CI (β)	β	95% CI (β)
ln DHEA (nmol/l)	0.013	−0.048 to 0.075	0.030^a^^,^ b	−0.029 to 0.088
ln DHEAS (µmol/l)	−0.029	−0.097 to 0.040	−0.003^a^^,^ c	−0.072 to 0.065
ln AD (nmol/l)	0.045	−0.0002 to 0.090	0.054* a ^,^ c ^–^ e	0.010 to 0.098
sqrt ADIOL (nmol/l)	0.022	−0.059 to 0.102	0.032^a^^–^ c ^,^ e	−0.047 to 0.110
ln E2 (pmol/l)	0.018	−0.020 to 0.056	0.025^c^	−0.013 to 0.063
ln E1 (pmol/l)	0.047*	0.004 to 0.090	0.048* c	0.005 to 0.092
ln E1S (nmol/l)	0.030	−0.047 to 0.108	0.037^d^^,^ g	−0.039 to 0.113
ln T (nmol/l)	−0.015	−0.057 to 0.027	−0.006^c^^,^ d	−0.048 to 0.036
sqrt fT (nmol/l)	−0.012	−0.025 to 0.002	−0.011^a^^,^ f	−0.023 to 0.002
sqrt BT (nmol/l)	−0.046	−0.098 to 0.005	−0.029^a^^,^ c	−0.079 to 0.021
ln DHT (nmol/l)	−0.051	−0.112 to 0.009	−0.031^a^^,^ c ^–^ f	−0.092 to 0.030
sqrt SHBG (nmol/l)	0.043	−0.136 to 0.222	0.010^a^^,^ d ^,^ f ^,^ h ^–^ j	−0.072 to 0.272
ln LH (IU/l)	0.064	−0.002 to 0.131	0.041^a^^–^ d ^,^ g	−0.029 to 0.110
ln FSH (IU/l)	0.071	−0.011 to 0.153	0.051^a^^–^ c ^,^ e	−0.035 to 0.136
sqrt T/LH	−0.071	−0.138 to −0.005	−0.037^a^^–^ c ^, ^ f ^–^ g	−0.106 to 0.031
ln T/E2	−0.032	−0.074 to 0.009	−0.036^c^^–^ e	−0.078 to 0.005

a : age;

b : alcohol;

c : season of blood sampling;

d : BMI;

e : education;

f : pp′-DDE;

g : smoking;

h : waist-to-hip-ratio;

i : blood total lipids;

j : chlordecone.

* Statistically significant association because 95% CI (β) does not include zero.

**Table 5:** Regression coefficients (β) for association between serum chlordecone levels and the outcomes variables.

Outcomes	Crude		Adjusted	
	β	95% CI (β)	β	95% CI (β)
ln DHEA (nmol/l)	0.001	−0.044 to 0.040	−0.005^a^^,^ b	−0.053 to 0.042
ln DHEAS (µmol/l)	−0.006	−0.058 to 0.046	−0.016^a^^–^>^d^	−0.069 to 0.036
ln AD (nmol/l)	0.020	−0.014 to 0.054	0.013^b^^–^ d ^,^ f ^,^ g	−0.021 to 0.048
sqrt ADIOL (nmol/l)	0.048	−0.013 to 0.108	0.041^c^^,^ d ^,^ g	−0.022 to 0.105
ln E2 (pmol/l)	0.003	−0.025 to 0.032	−0.011^a^^,^ c ^–^>^e^^,^ g ^,^ h	−0.018 to 0.041
ln E1 (pmol/l)	0.007	−0.026 to 0.039	0.016^b^^–^ d ^,^ h	−0.019 to 0.050
ln E1S (nmol/l)	0.014	−0.044 to 0.072	−0.014	−0.044 to 0.072
ln T (nmol/l)	0.014	−0.018 to 0.046	0.009^a^^,^ d ^,^ e	−0.025 to 0.042
sqrt fT (nmol/l)	−0.004	−0.014 to 0.007	−0.004^c^^,^ g ^,^ h ^,^ j	−0.005 to 0.013
sqrt BT (nmol/l)	0.0004	−0.040 to 0.041	−0.001^d^^,^ i ^,^ j	−0.042 to 0.039
ln DHT (nmol/l)	0.030	−0.016 to 0.075	0.027^a^^,^ b ^,^ d ^,^ e	−0.029 to 0.061
sqrt SHBG (nmol/l)	0.067	−0.064 to 0.198	0.061^b^^,^ d	−0.074 to 0.196
ln LH (IU/l)	−0.022	−0.071 to 0.025	−0.019^d^	−0.071 to 0.031
ln FSH (IU/l)	−0.023	−0.073 to 0.026	−0.018^c^	−0.082 to 0.045
sqrt T/LH	0.043	−0.020 to 0.106	0.059^c^^,^ d ^,^ i	−0.003 to 0.123
ln T/E2	0.012	−0.020 to 0.044	0.007^d^^,^ g	−0.025 to 0.040

a : education;

b : smoking;

c : age;

d : season of blood sampling;

e : BMI;

f : blood total lipids;

g : PCB-153;

h : pp′-DDE;

i : alcohol;

j : waist-to-hip-ratio.

We found a significant negative relationship between DDE and DHT concentration in both crude and adjusted models (β = −0.063, 95% confidence interval (CI) = −0.109 to −0.016, *p* = 0.008) and a positive relationship between DDE and LH in both crude (β = 0.053, CI = 0.001 to 0.106, *p* = 0.04) and adjusted (β = 0.057, CI = 0.005 to 0.109; *p* = 0.03) models (Table 3). T/LH ratio was negatively associated with DDE concentration in both crude (β = −0.066, CI = −0.118 to −0.013, *p* = 0.01) and adjusted (β = −0.061, CI = −0.112 to −0.010, *p* = 0.02) models.

PCB 153 concentration was positively associated to AD concentration at the limit of significance in the crude model (β = 0.045, CI = 0.0002 to 0.090, *p* = 0.05), and this relationship was significant in the adjusted model (β = 0.054, CI = 0.010 to 0.098, *p* = 0.02). PCB 153 concentration was also significantly and positively associated with E1 levels in the crude (β = 0.047, CI = 0.004 to 0.090, *p* = 0.03) and adjusted (β = 0.048, CI = 0.005 to 0.092, *p* = 0.03) models (Table 4).

No association was observed between chlordecone and any outcome in either the crude or the adjusted model (Table 5).

## Discussion

We investigated associations between POPs with endocrine activities and the levels of a large panel of hormones involved primarily in the steroid pathway, in middle-aged French West Indian men.

As expected and found in most populations worldwide, DDE and PCB congeners 138, 153 and 180 were the most prevalent POPs found in the blood in our population. Moreover, blood concentrations of these pollutants are in the range of background environmental levels currently found in US populations of similar age range [37]. This is not surprising because the French West Indies has and has had only very limited industrial activities involving significant use or emission of PCBs. The use of DDT in agricultural supplies or for disease vector control was anecdotic. Consequently, exposure to these chemicals is likely to be associated with background contamination of the food chain. The only POPs that have been spread in French West Indies were technical grade HCH, a mixture of α, β, and γ isomers, mainly before 1970, and chlordecone, intensively from 1973 to 1993; both were used to control the banana root borer. Unlike HCH, chlordecone undergoes no significant biotic or abiotic degradation in the environment, and permanently polluted soils and waterways has been and are still nowadays the major source of human contamination in French West Indies, through the consumption of contaminated foodstuffs [38]. The occupational profile of our population study reflects that of the general male Guadeloupean population aged from 45 to 70 years old. Among the 277 men included in the study, only 29 are or have been workers on banana farms and only 15 of these were occupationally exposed to chlordecone during the period 1973–1993 (data not shown). This unique situation provided us with an opportunity to study the effects of three prevalent POPs with different endocrine modes of action, at environmental exposure levels.

Our study population was randomly selected from among the general population of Guadeloupe and we excluded any subjects with abnormalities or medical conditions that may alter systemic levels of steroid hormones. As a consequence, the values reported may be considered as the normal range of values for the local male population and for the age range investigated (45–70 years old) [29].

Our findings provided some evidence of significant dose-dependent effects. However, the association between exposure and outcome differed between pollutants. DDE exposure levels were negatively associated to DHT levels and positively associated to LH concentration. Levels of PCB 153 were positively associated to AD and E1 concentrations. By contrast, no association was found between chlordecone exposure and any of the steroids, binding proteins or gonadotrophins investigated.

Our findings are consistent with most previous studies, regardless of the range of age and exposure levels considered, showing an absence of association between the levels of DDE and PCB 153 and those of T, E2, SHBG or FSH. However, some conflicting results have been published, including lower testosterone levels with increasing PCB 153 levels in Native American men aged 18 to 88 [17], lower E2 levels with increasing DDE levels in Swedish fisherman aged 18 to 45 [15] and in Thai men aged 48 to 82 [13], and higher SHBG levels with increasing DDE and PCB 153 concentrations in Ukrainian men aged 19 to 45 years [14]. Haugen et al. [19] also reported an increase in SHBG concentration with increasing PCB 153 levels, but not with increasing DDE levels, in Norwegian men aged 19 to 40.

This study differs from previous studies in the field in several ways. Major changes in blood steroid hormone levels occur during adulthood, and studies covering broad age ranges, from early to late adulthood may be inappropriate. By restricting our investigation to the 45–69 year age range, we focused on a period in which hormonal imbalance is known to play a key role in some aging-related diseases. Other strengths of this study include the simultaneous determination of serum concentrations of a large number of steroid hormones, mostly by GC–MS. This sensitive and specific method is currently considered the most accurate approach to the determination of steroid hormones [26], [27]. We excluded obese subjects and those with acute or chronic pathological conditions thought to have a strong impact on hormone metabolism. We also collected extensive data for adjustment on the basis of factors associated with changes in blood steroid hormone levels and to reduce the bias of the estimates.

Our cross-sectional study also presents several limitations. Given the years during which DDT and PCBs were used worldwide, the study population had been exposed to these chemicals or their metabolites throughout much of their lifetimes. For chlordecone, the exposure period began in 1973, at a median age of 30 years. Single blood determinations to estimate exposure to these contaminants may not adequately reflect past exposure. However, unlike women, men are not subject to the mobilization of fat-soluble pollutants during pregnancy or breastfeeding that can significantly alter the pollutant load of the whole body. Any previous weight loss or gain, particularly if large, may modify the blood concentration of these pollutants, but we believe that this factor had a weak influence overall in our free-chronic disease and non-obese population study. Consequently, in adult healthy males, and for chemicals with long half-lives in the body, single blood determinations may be considered a satisfactory surrogate of past exposure. Finally, we cannot exclude the possibility of confounding with other undetermined chemicals, due to a lack of consideration of interactions between chemicals or factors known to influence blood steroid levels, such as diet and physical activity, or chance findings.

We focused on steroid hormone levels in the general circulation, but the results obtained may not be entirely representative of what occurs in target tissues. Steroid hormones may act on tissues where they are synthesized or be secreted into the blood and act remotely on other target tissues [39], [40]. Also, reduced T metabolites (DHT) which are produced locally in target tissues may reenter the general circulation. Endocrine-disrupting chemicals may exert endocrine effects by a multitude of mechanisms that are not yet fully understood and the pathways altering steroid hormone levels may be unpredictable.

DDE has antiandrogenic effects in vivo, as assessed from changes in the weights of androgen-responsive tissues [41]. Such effects are likely to be mediated by inhibition of AR binding and/or AR-dependent gene expression [23], [42]. These properties cannot directly explain the relationship between increased DDE exposure and decreased serum DHT levels observed in our study population. However, our findings provide a different type of confirmation of the antiandrogenic properties of DDE. Interestingly, Lo et al [43] have shown that DDE is able to inhibit 5α-reductase in human prostate tissue homogenates. Because serum T did not seem to be affected by DDE exposure and 5α-reductase is involved in the conversion of T to DHT, it is plausible that decreased serum DHT levels may be explained by 5α-reductase inhibition. High levels of DDE also appeared to be associated with high levels of LH. This finding may be related with the feedback provided by circulating DHT, directly at the pituitary and centrally at the level of the hypothalamus [44].

Many of the known endocrine activities of PCBs involve steroid hormone signaling systems including estrogens, androgens, progestins and adrenal steroids [45]. Individual congeners may have different, and in some cases even opposite effects. Some PCBs have estrogenic activities, whereas others are antiestrogenic. The most prevalent non dioxin-like PCBs (138, 153, and 180) have been reported to be antiestrogenic in both reporter gene and MCF-7 cell proliferation assays [46], [47] and to decrease ER-mediated activity in ER-CALUX bioassays (24). These studies led to suggestions that these compounds might decrease the amounts of E2 in the circulation [24], [47]. However, other authors [48], based on the observation that various environmentally relevant PCB-hydroxylated metabolites are potent inhibitors of human estrogen sulfotransferase, have suggested that PCBs may increase E2 bioavailability in target tissues. Here, consistent with most previous studies on adult men, we observed no change in blood E2 concentration as a function of non dioxin-like PCB levels. By contrast, we found that PCB exposure was associated with high levels of AD in the blood, an observation not previously reported in humans. However, high levels of AD production have been reported in the liver and testis of bulls chronically exposed to PCBs [49]. There are two major pathways for the production of AD from pregnenolone, one involving DHEA and the other, progesterone. Because DHEA levels are not modified by PCBs exposure, we can hypothesize that increased AD levels may have its origin in any upstream step arising from the progesterone pathway. The positive association between PCB 153 and E1 may result from an increase in AD precursor levels, consistent with mass action rather than an increase in aromatase activity.

Chlordecone binds ERα and ERβ, acting as an agonist of ERα and an antagonist of ERβ [25], [50]. In addition to its interaction with nuclear ER, chlordecone may activate alternative estrogen signaling pathways or other enzymes and receptors involved in steroid homeostasis [51]–[54]. However, such modes of action did not appear to affect circulating steroid levels in our population study. Few studies have addressed the issue of the effect of chlordecone exposure on the blood levels of steroid hormones or gonadotrophins. Subchronic exposure of male rats to chlordecone do not affect serum testosterone levels [55]. When primary rat pituitary cell cultures were treated with chlordecone, basal secretion of FSH and LH were not altered [56]. To our knowledge, there have been no studies on humans to investigate the effects of chlordecone on circulating levels of steroid hormones, binding proteins or gonadotropins. Here, as in a preliminary study we carried out in 100 healthy male subje[ts aged 20 to 50 years [57], we found no significant association between chlordecone exposure at environmental levels and the concentrations of steroids, binding proteins or gonadotrophins investigated.

The ethnic origin of study populations must also be taken into account. Most previous studies were carried out on men of European descent, whereas our study population was of African descent. We recently reported differences in the concentrations of certain steroids in the blood (particularly AD and E1) between our study population and healthy European men of similar age [29]. This may be explained by different levels of transcription or allele frequency of polymorphisms of steroid hormone related genes [58]. Consequently, we cannot exclude that EDCs effects on steroid hormone levels may be at least partially ethnically sensitive.

In summary, in this cross-sectional study performed in middle-age men, we found that the pattern of endocrine response, estimated by blood levels of steroid hormones, to long-term exposure of POPs with endocrine activity varies depending on the pollutant studied. Such differential pattern could be related to the different modes of action that are usually attributed to them. It remains unclear whether such changes in circulating steroid hormone levels are predictive of the subsequent occurrence of disease.

## Supporting Information

Supporting Information File S1 **Supporting tables.**
(DOC)
