# Supplementary material for: Persistent Organochlorine Pollutants with Endocrine Activity and Blood Steroid Hormone Levels in Middle-Aged Men
Source: PLoS One. 2013 Jun 13;8(6):e66460. doi: 10.1371/journal.pone.0066460 (PMC3681943; doi:10.1371/journal.pone.0066460)
Supplement: Supporting Information File S1 — Supporting tables. (DOC) [file pone.0066460.s001.doc]

**Supplemental table 1**

Hormone quality controls

| **Assay method** | **Analytes** | **Target ions analyte / IS**  **(amu)** | **Range of detection** ^(a)^ | **Quality Controls (QC)** | | | |
| --- | --- | --- | --- | --- | --- | --- | --- |
|  |  |  |  | **Lower limit of quantification** | **Low QC** | **Middle QC** | **High QC** |
|  |  |  |  | Mean ^(a)^, Intra-assay coefficient of variation (%), Inter-assay coefficient of variation (%) | | | |
| GC-MS ^(c)^ | DHEA | 482 / 485 | 0.64 - 28.09 | 0.64, 5.4, 6.1 | 2.60, 3.5, 4.7 | 6.90, 2.6, 2.8 | 17.58, 1.4, 2.0 |
|  | AD | 461 / 468 | 0.17 - 14.14 | 0.17, 6.2, 10.8 | 1.68, 3.7, 4.9 | 3.53, 3.1, 4.1 | 7.05, 2.4, 3.8 |
|  | ADIOL | 678 / 683 ^(b)^ | 0.37 - 13.95 | 0.37, 7.0, 11.0 | 1.06 4.0, 4.4 | 2.55, 3.7, 4.2 | 5.10, 2.7, 3.2 |
|  | T | 482 / 485 | 0.17 - 28.09 | 0.17, 4.8, 10.1 | 1.66, 2.2, 3.1 | 8.53, 2.1, 2.5 | 20.60, 1.0, 1.7 |
|  | E1 | 464 / 468 | 29.1 - 2996 | 29.1, 7.4, 9.5 | 53.3, 4.3, 4.7 | 288.1, 3.1, 3.3 | 735.6, 2.6, 3.0 |
|  | E2 | 660 / 664 | 7.7 - 1487 | 7.7, 8.9, 10.5 | 36.0, 3.5, 4.1 | 176.2, 2.6, 3.0 | 728.0, 1.5, 1.7 |
|  | E1S | 464 / 468 ^(d)^ | 0.13 - 16.11 | 0.13, 8.7, 11.0 | 1.34, 4.0, 4.4 | 4.00, 3.1, 3.4 | 7.98, 2.8, 2.9 |
|  | DHT | 484 / 487 | 0.07 - 13.95 | 0.07, 7.8, 9.0 | 0.65, 3.0, 3.1 | 1.65, 2.0, 2.3 | 5.13, 1.7, 1.7 |
| Ammonium sulfate precipitation + GC-MS | BT |  |  |  | 1.01, 3.2, 3.5 | 3.74, 4.2, 4.3 | 8.98, 1.4, 1.5 |
| RIA | SHBG |  | 6.0 - 164.0 |  | 16.0, 2.0, 2.3 | 41.9, 2.9, 3.3 | 68.4, 1.8, 2.3 |
|  | DHEAS |  | 0.23 - 23.05 |  |  | 1.4, 2.2, 3.5 |  |
|  | FSH |  | 0.5 - 65.0 |  | 8.8, 4.0, 4.2 | 27.3, 2.8, 3.0 | 48.1, 2.1, 2.2 |
|  | LH |  | 0.5 - 54.0 |  | 5.9, 7.0, 7.6 | 21.6, 2.8, 3.1 | 42.8, 3.5, 3.7 |

^a^: in nmol/l, except E2 and E1 in pmol/l, DHEAS in µmol/l, and FSH and LH in mIU/ml.

^b^: internal standard, 3β,diol,d_3_ (5α,androstane,3β, 17β,diol,d_3_, 683 amu).

^c^: accuracy: -3.8 to +3.4 %

^d^: after solvolysis

**Supplemental table 2**

Spearman’s rank correlation analysis of the relationship between levels of frequently detected (LD > 80 %) pollutants

|  | **p,p’-DDE** | **PCB 138** | **PCB 153** | **PCB 180** | **Chlordecone** |
| --- | --- | --- | --- | --- | --- |
| **p,p’-DDE** |  | 0.39  *p* < 0.0001 | 0.30  *p* < 0.0001 | 0.23  *p* = 0.0003 | 0.02  *p* = 0.75 |
| **PCB 138** |  | **-** | 0.89  *p* < 0.0001 | 0.88  *p* < 0.0001 | 0.03  *p* = 0.63 |
| **PCB 153** |  | **-** | **-** | 0.83  *p* < 0.0001 | - 0.03  *p* = 0.59 |
| **PCB 180** |  | **-** | **-** | **--** | 0.01  *p* = 0.84 |
| **ΣPCB (138 +153+180)** | 0.31  *p* < 0.0001 |  | **-** | **-** | 0.004  *p* = 0.84 |

Values < limits of detection (LD) were estimated by the maximum likelihood estimation method (Jin Y, Hein MJ, Deddens JA, Hines CJ. 2011. Analysis of lognormally distributed exposure data with repeated measures and values below the limit of detection using SAS. Ann Occup Hyg 55: 97-112).
